# MLKL is involved in the regulation of skin wound healing and interplay between macrophages and myofibroblasts in mice

**DOI:** 10.1038/s41598-025-97729-2

**Published:** 2025-04-19

**Authors:** Jiamin Zhao, Shuangyi Zhang, Yunjie Bai, Zhiguo Gong, Wenhui Bao, Zhuoya Yu, Bo Liu, Wei Mao, Surong Hasi

**Affiliations:** 1 Key Laboratory of Clinical Diagnosis and Treatment Techniques for Animal Disease, Ministry of Agriculture, Inner Mongolia Agricultural University, No. 29, Erdosdong Road, Saihan District, Hohhot, 010011 China; 2 Laboratory of Veterinary Clinical Pharmacology, College of Veterinary Medicine, Inner Mongolia Agricultural University, Hohhot, China

**Keywords:** Skin, Wound healing, MLKL, Macrophages, Myofibroblasts, Immunology, Molecular biology

## Abstract

Mixed lineage kinase domain-like protein (MLKL), a critical necroptosis effector, is strongly linked to inflammation, a key component of skin wound healing. However, its precise role in the wound healing process remains inadequately characterized. This study revealed sustained MLKL overexpression throughout the wound healing process, not limited to the early inflammation phase. Wound healing was delayed in MLKL-deficient (MLKL^−/−^) mice compared to wild type C57BL/6J (MLKL^+/+^) mice, with impaired morphological and pathological recovery. MLKL deficiency reduced the synthesis of inflammatory factors (IL-6, TNF-α, PGE_2_), tissue repair molecules (EGF, VEGF, ERα, MMP-9), and apoptosis markers (Caspase-3, p53, Bcl-2) at wound site. Subsequently, a co-culture system was established to explore the roles of MLKL in macrophage-fibroblast interactions. M1 or M2 macrophages (M1ø or M2ø) were co-cultured with fibroblast-conditioned medium (MFbCM), and fibroblasts were co-cultured with macrophage-conditioned medium (M1ø CM or M2ø CM). The results indicated that MLKL^+/+^ M1ø CM and M2ø CM significantly increased ERα, VEGF and MMP-9 protein expression levels in fibroblasts, whereas this effect was impaired when MLKL^−/−^ M1ø CM or M2ø CM were used. Similarly, MLKL^+/+^ MFbCM upregulated IL-6, NO, and TNF-α in M1ø and IL-10, arginase, and Ym-1 in M2ø, but these effects were diminished with MLKL^−/−^ MFbCM treatment. These results indicate bidirectional crosstalk between macrophages and fibroblasts, in which MLKL plays a role. Additionally, PGE_2_ was identified as a downstream mediator of MLKL-mediated interactions between macrophages and fibroblasts. In conclusion, these findings identify MLKL as a multifunctional regulator in skin wound healing in mice.

## Introduction

Skin, the largest organ in the body, safeguards normal physiological activities and protects internal organs from environmental, mechanical, microbial, and chemical stimuli. The skin frequently experiences wounds when exposed to various external stimuli. An evolutionarily conserved process exists for wound closure, including hemostasis, inflammation, cellular proliferation, and remodeling^1^. Wound healing is typically regulated by a complex system of chemokines, cytokines, and tissue growth factors, along with the interactions between different cell types at the wound site^2^. Impairments or delays in any step of these processes can result in prolonged wound healing. Thus, a comprehensive understanding of the skin wound healing process at the molecular level is essential to enhance therapeutic strategies for wound management.

When tissues sustain mechanical injury, an inflammatory response is triggered by damage-associated molecular patterns (DAMPs) released from dead and dying cells^3^. Cell death is a critical biological process that is essential for restoring and maintaining skin homeostasis. It also facilitates recovery from acute injuries and infections by regulating the barrier function and immune responses. Lytic forms of cell death, characterized by the loss of plasma membrane integrity, can provoke inflammation due to the uncontrolled release of intracellular contents. Programmed cell death occurs in several distinct forms, including apoptosis, necroptosis, pyroptosis, NETosis, and ferroptosis, many of which contribute to inflammation and associated diseases. Apoptosis involves a caspase cascade to minimize inflammation by preventing the activation of inflammatory pathways within the cell and reducing the immunogenicity of cellular contents. In contrast, necroptosis proceeds independently of caspases and involves permeabilization of the plasma membrane and early lysis, thus lacking the protective mechanisms that limit immunogenicity. This inflammatory process releases intracellular components that function as DAMPs^4^. Elevated levels of receptor-interacting protein kinase 3 (RIPK3) and pseudokinase mixed lineage kinase domain-like proteins (MLKL) are associated with a necrosome complex formation. Within this complex, RIPK3 interacts with the RHIM domain of RIPK1, where phosphorylation stabilizes its association and activates RIPK3^5–7^. Phosphorylated RIPK3 subsequently phosphorylates MLKL, which oligomerizes and translocates to the plasma membrane. This disrupts the membrane, leading to the release of intracellular contents, cellular swelling, rupture, and necroptotic cell death^8–10^. Although necroptosis is closely related to inflammation, which is an essential process in skin wound healing, there is currently no convincing evidence that necroptosis or related effector (e.g., MLKL) participates in skin wound healing.

After injury, macrophages are recruited to the wound site after or simultaneously with neutrophil invasion^2^. Macrophages are highly efficient in tissue repair because of their versatility and plasticity^11^. They phagocytose necrotic cellular debris and pathogenic materials or microorganisms at the site of injury via evolutionarily conserved receptors. Macrophages exhibit morphological changes and other actions in response to local signals^2^. Traditionally, macrophages have been categorized into two main subsets: classically (M1) and alternatively (M2) activated. M1 macrophages are induced by pro-inflammatory stimuli and further propagate inflammation by releasing inflammatory cytokines (e.g., interleukin-1(IL-1), IL-6, and tumor necrosis factor-α (TNF-α)). As wound healing progresses, these macrophages phagocytose apoptotic neutrophils, replacing them with primary inflammatory cells. During the later stages of wound healing, M2 macrophages release anti-inflammatory mediators and growth factors that promote angiogenesis, reepithelialization, and fibroplasia etc^12^. The ablation of macrophages leads to delayed wound repair, directly demonstrating their crucial role in wound healing^13^. Similarly, delayed re-epithelialization and angiogenesis were observed when macrophages were knocked down during the early healing stages^2^. Macrophages in the wound bed activate proliferation in fibroblasts^14^. After injury, multiple fibroblast subsets become activated myofibroblasts, contributing to tissue repair and scar formation^14^. Fibroblasts produce extracellular matrix (ECM) molecules that regulate the tissue strength and resilience. An imbalance in ECM maintenance can lead to tissue dysfunction^15^.

Although the individual roles of macrophages and fibroblasts in skin wound healing are well defined, the crosstalk between these cells is crucial in the four overlapping phases of wound healing, and the underlying mechanisms have not yet been fully elucidated. Miscommunication between macrophages and fibroblasts is a critical factor that shifts the balance from physiological repair to pathological fibrosis^15^. Macrophage depletion during wound healing leads to a reduction in the number of fibroblasts^16^. Multiple cytokines are potential mediators in the interaction between macrophages and fibroblasts, including fibroblast growth factors (FGFs), platelet-derived growth factor, vascular endothelial growth factors (VEGFs), IL-6, IL-13, prostaglandin E_2_ (PGE_2_), and transforming growth factor-β (TGF-β1) etc^17–20^. In a cutaneous wound model, the expression of cyclooxygenase-2/PGE_2_/EP4 cascade increases after damage^21,22^. Elevated PGE_2_ level accelerates the cutaneous wound healing process^21,23^. PGE_2_ not only accelerates the healing rate but also remodels the skin structure at injured sites with new hair follicles and sebaceous glands. Furthermore, PGE_2_ hydrogels display obvious anti-inflammatory and pro-angiogenic effects by inducing macrophage polarization from the M1 phenotype to M2 phenotype at the injured sites. Previous studies have shown that PGE_2_ affects the migration and contraction of human fetal dermal fibroblasts, preventing fibrotic processes during wound healing^24,25^. Myofibroblast-derived PGE_2_ activates macrophages by interacting with specific receptors^20^. These phenomena indicate that PGE_2_ might be a potential intermediatory in mediating the interaction between macrophages and fibroblasts in wound healing process, which needed to be clarified.

In this study, wild type C57BL/6J (MLKL^+/+^) and MLKL-deficient (MLKL^−/−^) mice were used as experimental models to examine the role of MLKL in skin wound healing by creating dorsal cutaneous wounds. The role of MLKL in the interaction between macrophages (M1 and M2) and myofibroblasts was also investigated. Furthermore, we identified a potential intermediary, PGE_2_, that is responsible for mediating the role of MLKL in regulating the interaction between M1/M2 macrophages and myofibroblasts.

## Results

### MLKL expression and its impact on skin wound healing in mice

**Fig. 1:**
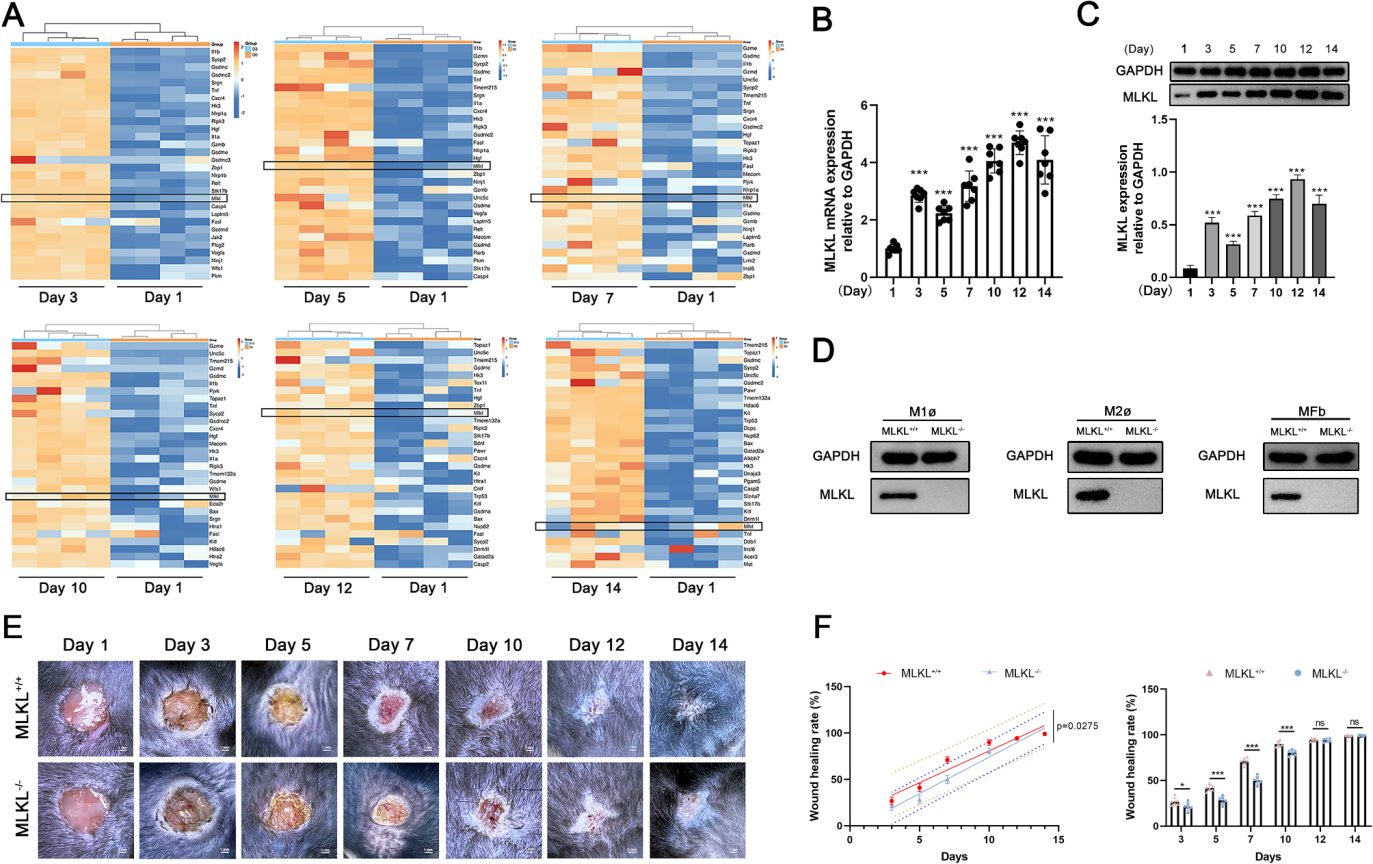
MLKL expression and its role in skin wound healing dynamics in mice. (**A**) Heatmap shows top up-regulation 30 genes from programmed cell death in wound tissues from day 1 to 14. Horizontal represents genes, where each column represents one sample. Red represents increased expression genes, and blue represents decreased expression genes (*n* = 4). (**B**) MLKL mRNA expression in MLKL^+/+^ skin wound tissues from day 1 to 14 was measured by RT-PCR (*n* = 7). GAPDH was used as a loading control. (**C**) MLKL protein expression in MLKL^+/+^ skin wound tissues from day 1 to 14 was measured by western blot (*n* = 4). GAPDH was used as a loading control. Grayscale values were measured using ImageJ software. (**D**) Western blot analysis of MLKL protein levels in M1 macrophages, M2 macrophages and myofibroblasts obtained from MLKL^+/+^ mice and MLKL^−/−^mice. (**E**) The 6 mm diameter excisional biopsies were obtained from the back of MLKL^+/+^ mice and MLKL^−/−^ mice. (**F**) Linear regression analysis was done with day as independent variable and reduction in wound area (healing rate) as dependent variable. The mean value of wound healing rate was calculated based on the original wound size of each biopsy site (*n* = 6). Littermate control mice were on a C57BL/6J genetic background were utilized in the experiments. Results were expressed as the mean ± SD and analyzed by Student t test or one-way ANOVA followed by Tukey’s multiple comparisons test. *P < 0.05, ***P < 0.001. *ns* not significant. Day 1 refers to the day the injury was created.

To identify potential target genes associated with tissue repair, particularly those related to cell programmed death, we conducted transcriptome sequencing at the wound site in MLKL^+/+^ mice from day 1 to 14. Based on transcriptome sequencing data, we selected genes associated with cell programmed death and mapped the top 30 upregulated genes. MLKL were identified with sustained overexpression throughout the wound healing process from day 1 to 14 (Fig. 1A). The mRNA and protein expression confirmed that MLKL was consistently overexpressed in the wound tissue from day 1 to 14 (Fig. 1B, C). A specific anti-MLKL antibody was used to verify the absence of MLKL protein in M1 macrophage, M2 macrophage, and myofibroblast obtained from MLKL knockout mice (Fig. 1D). Collectively, these findings suggest that MLKL may serve as a potential regulator of skin wound healing in mice.

**Fig. 2:**
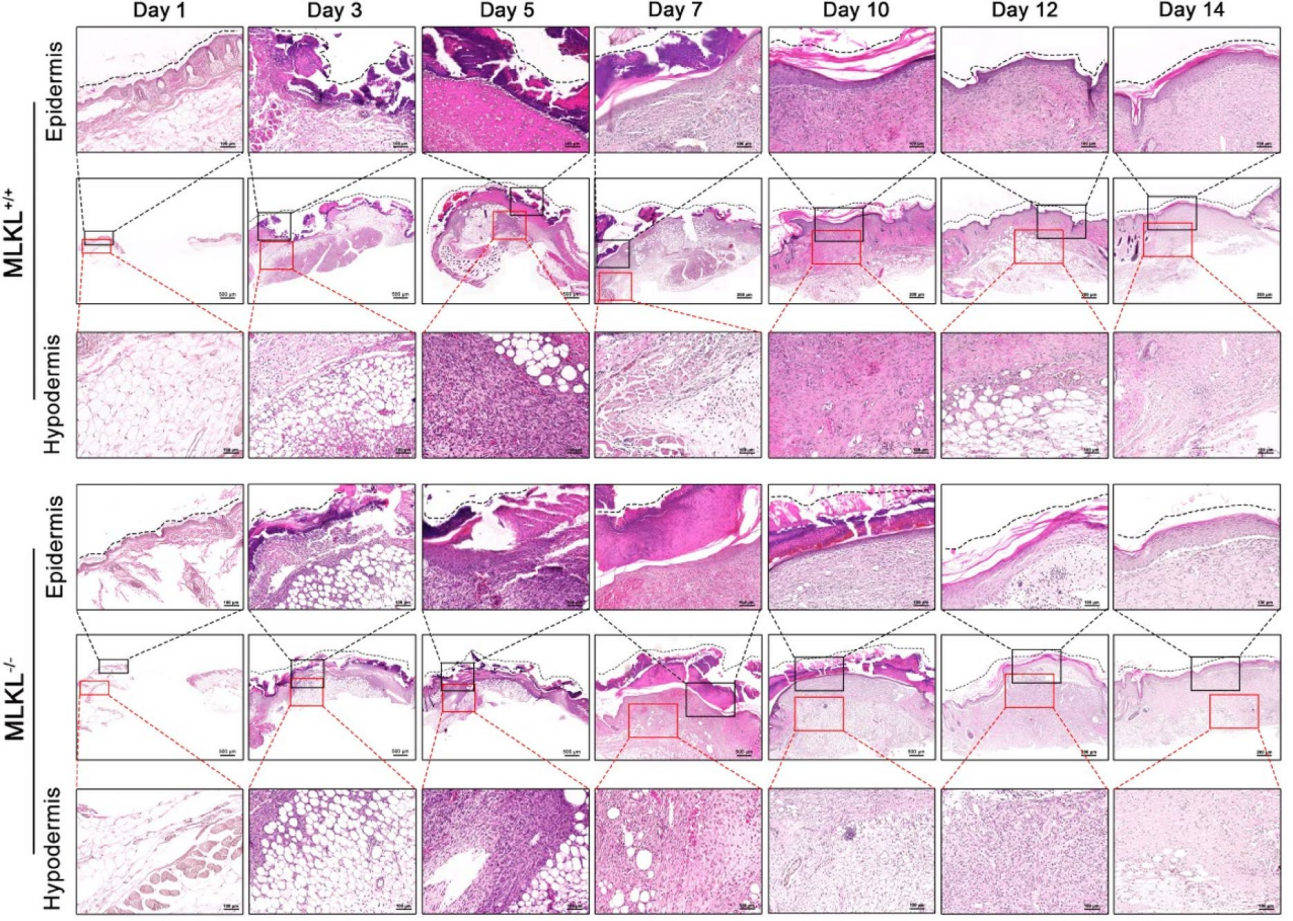
Delay of skin wound healing in MLKL^−/−^ mice. The representative histological examination of MLKL^+/+^ and MLKL^−/−^ mice skin wound by H&E staining at day 1, 3, 5, 7, 10,12 and 14 after injury. Littermate control mice were on a C57BL/6J genetic background were utilized in the experiments. The wound surface was marked by dashed line. Scale bar = 500, 200 or 100 μm. Day 1 refers to the day the injury was created.

To investigate the involvement of MLKL in skin wound healing, the process of skin wound closure was compared between MLKL^+/+^ and MLKL^−/−^ mice within 14 days. The delayed wound healing was observed in MLKL^−/−^ mice compared with that in MLKL^+/+^ mice, which was obvious on days 3, 5, 7, and 10 (Fig. 1F). The wound healing ratios are shown in (Table 1). The histological characteristics of the wound closure process indicated that the healing speed of skin wounds was impaired in MLKL^−/−^ mice. On day 3, necrotic scabs were observed on the surface of the wounds of both mice, with a large amount of serous fluid, cellulose exudation, and granulation tissue regeneration at the edge of the wounds (Fig. 2). On day 5, the surface of the wounds in both groups was covered with a thick layer of necrotic scab, and the granulation tissue filled the wounds and began to mature, with epidermal differentiation and thickening. The difference was that more granulation tissue filled the wounds of MLKL^+/+^ mice than those of MLKL^−/−^ mice, and epidermal regeneration covered half of the wounds in MLKL^+/+^ mice and 1/3 of the wounds in MLKL^−/−^ mice (Fig. 2). On day 7, the epidermis on the wound surface of the two types of mice had completely regenerated, forming a thick layer covering the wound. The wound was filled with regenerated granulation tissue, and most of the granulation tissue was mature. The difference was that the granulation tissue near the regenerated epidermis in the wounds of MLKL^+/+^ mice was clearly congested. Local granulation tissue was congested between granulation tissue and epidermal regeneration in the wounds of MLKL^−/−^ mice (Fig. 2). On day 10, granulation tissue filled the wounds in both types of mice, most of which were mature. The number of collagen fibers in the wounds of MLKL^+/+^ mice was significantly higher than that in the wounds of MLKL^−/−^ mice (Fig. 2). On day 12, granulation tissue filled the wounds of both types of mice, and the epidermis regenerated completely and differentiated well, forming a thick epidermis. Most of the granulation tissue in the wounds of the MLKL^+/+^ mice was mature, with increased collagen fibers and local vascular congestion. Most granulation tissues in the wounds of MLKL^−/−^ mice began to mature and scattered neutrophils infiltrated the granulation tissue (Fig. 2). On day 14, the granulation tissue in the wounds of both mice was filled and fully mature, and epidermal regeneration completely covered the wounds to form a thick layer (Fig. 2). These findings suggested that MLKL is involved in skin wound healing in mice.

**Table 1:** Wound healing ratio.

Wound healing						
Day	MLKL^+/+^			MLKL^−/−^		
	Mean (%)	±SD	*n*	Mean (%)	±SD	*n*
3	26.87	3.91	6	21.43	4.12	6
5	41.11	3.95	6	28.17	3.99	6
7	71.01	3.32	6	50.12	4.18	6
10	89.98	2.81	6	80.44	2.79	6
12	94.47	1.45	6	93.98	1.71	6
14	99.20	0.29	6	99.12	0.55	6

### MLKL deficiency impairs inflammatory response and apoptosis in wound tissue

**Fig. 3:**
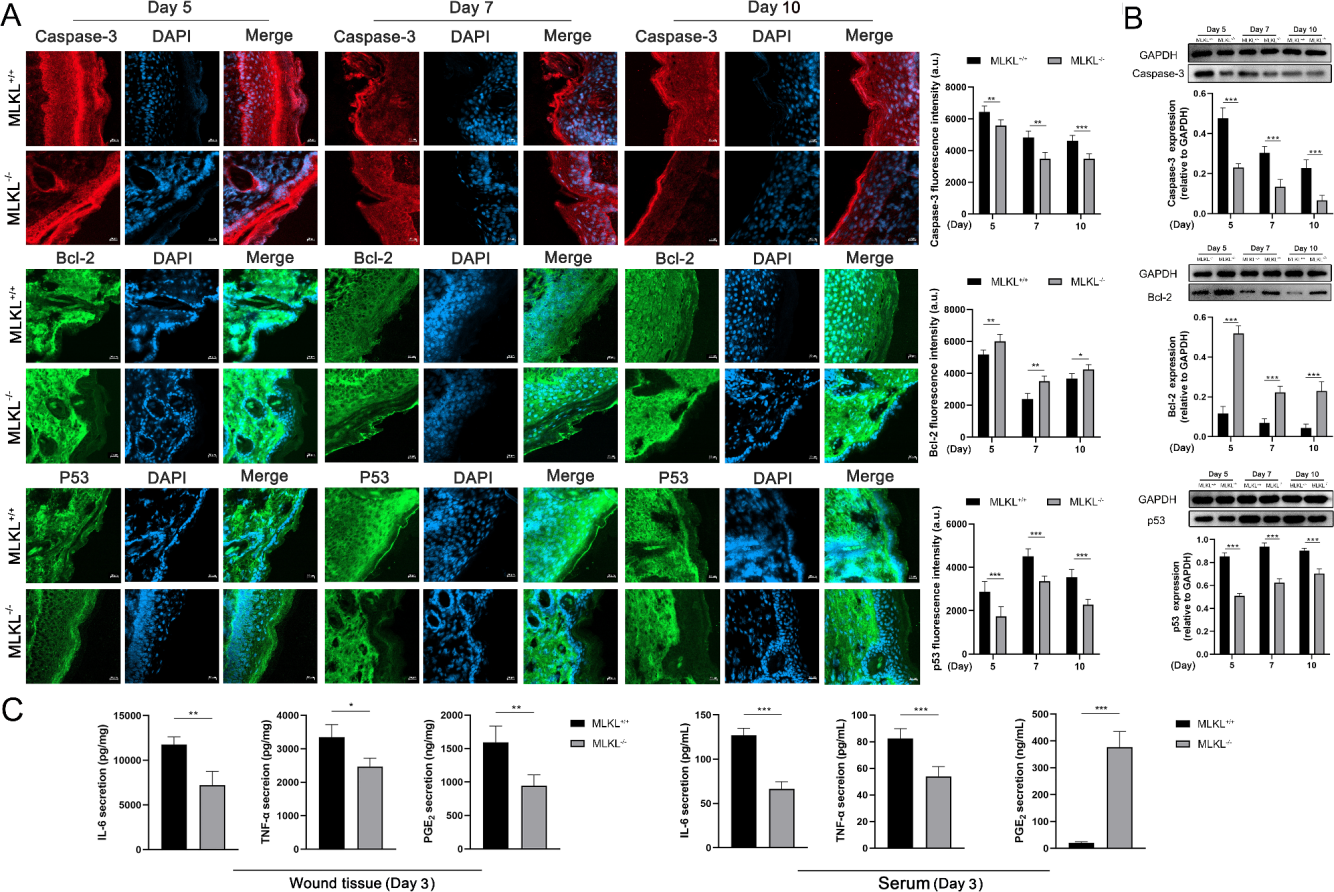
MLKL is involved in inflammatory mediator synthesis and apoptosis in wound site. (**A**) The skin wound tissues of MLKL^+/+^ and MLKL^−/−^ mice were subjected to immunofluorescence staining for Caspase-3 (red), Bcl-2 (green) P53 (green) and nuclear (DAPI, blue) on the 5, 7 and 10th days after wound injury. Merge represents the composite picture of target protein and nuclear. The confocal microscope was used for image acquisition (×200 magnification), and the mean value of fluorescence intensity was used to perform calculation (*n* = 6). (**B**) The protein expression of Caspase-3, Bcl-2 and P53 in wound tissue of MLKL^+/+^ and MLKL^−/−^ mice was evaluated by western blot (*n* = 4–6). GAPDH was used as a loading control. Grayscale values were measured using ImageJ software. (**C**) The secretion of IL-6, TNF-α and PGE_2_ in wound tissue and serum of MLKL^+/+^ and MLKL^−/−^ mice was evaluated by ELISA (*n* = 6). Littermate control mice were on a C57BL/6J genetic background were utilized in the experiments. Results were expressed as the mean ± SD of multiple independent experiments and analyzed by Student t test. *P < 0.05, **P < 0.01, ***P < 0.001. *a. u.* arbitrary unit. Scale label = 20 μm.

The inflammatory mediators and apoptosis related factors were detected in wound tissue of MLKL^+/+^ and MLKL^−/−^ mice. The expression levels of caspase-3 and p53 in the wound area of MLKL^−/−^ mice were lower than those in MLKL^+/+^ mice, as determined by immunofluorescence staining and Western blot analysis. (Fig. 3A, B). The expression of B cell leukemia 2 (Bcl-2) in the MLKL^−/−^ wound area was higher than that in the MLKL^+/+^ mice (Fig. 3A, B). Second, the inflammatory mediator including IL-6, TNF-α, and PGE_2_ secretion in MLKL^−/−^ wound tissue was lower than that in MLKL^+/+^ mice (Fig. 3C). For serum, IL-6 and TNF-α secretion in MLKL^−/−^ mice was lower than in MLKL^+/+^ mice. PGE_2_ levels in the sera of MLKL^−/−^ mice were higher than those in the sera of MLKL^+/+^ mice. In addition. the pre-injury PGE_2_ level in serum of MLKL^+/+^ and MLKL^−/−^ mice was approximately 0.4 ng/mL, as determined by ELISA. After injury, the PGE_2_ levels in serum of MLKL^+/+^ and MLKL^−/−^ mice significantly increased compared to pre-injury (Supplementary Material, Fig. S1). Taken together, these results suggest that MLKL is involved in the regulation of apoptosis and inflammatory responses during the wound healing process.

### MLKL deficiency impairs tissue growth factor expression in wound site

**Fig. 4:**
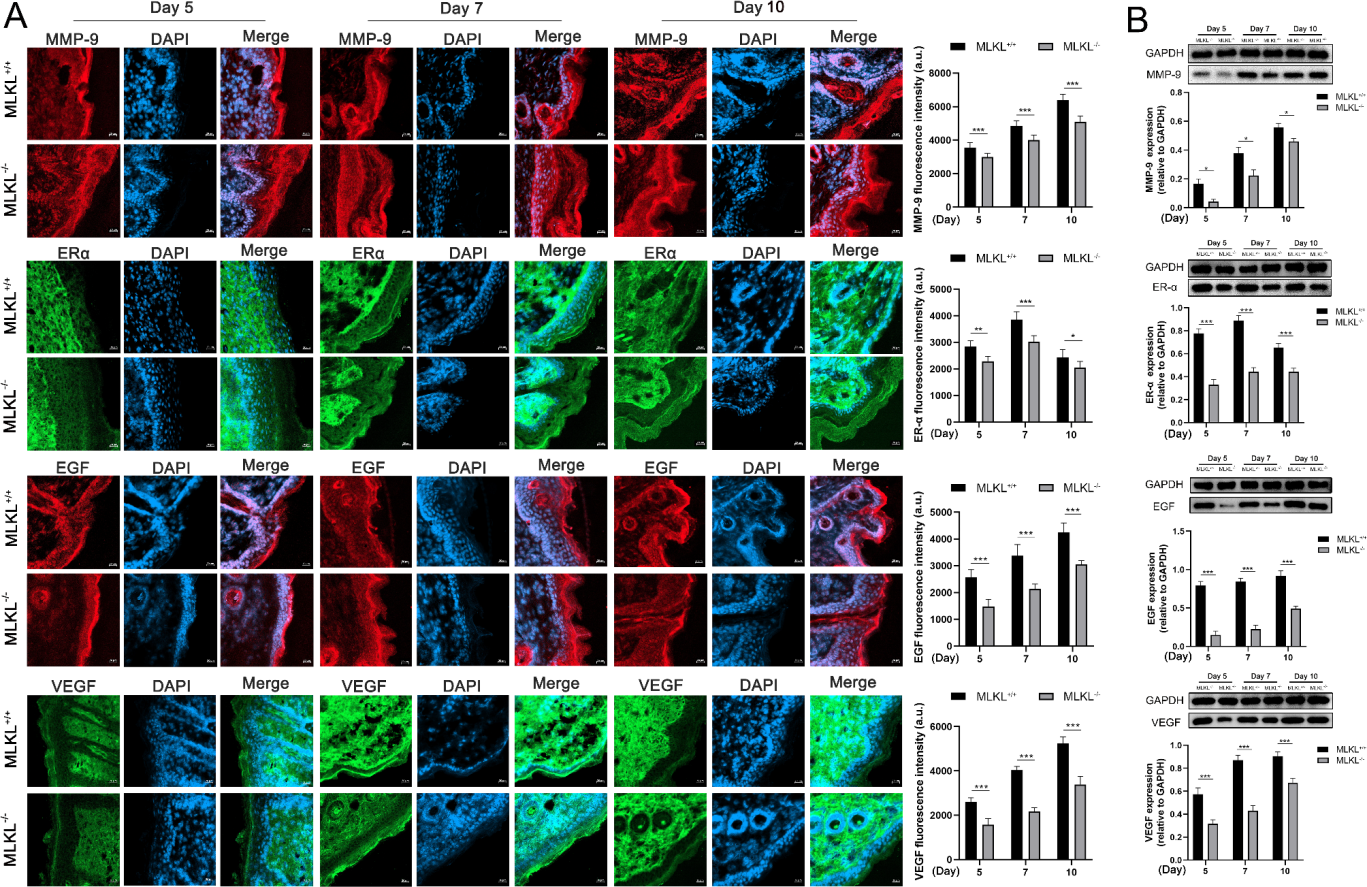
MLKL is involved in tissue growth related factors expression in wound site (**A**) The skin wound tissues of MLKL^+/+^ and MLKL^−/−^ mice were subjected to immunofluorescence staining for MMP-9 (red), ERα (green), EGF (red) VEGF (green) and nuclear (DAPI, blue) on the 5th, 7th and 10th days after wound injury. Merge represents the composite picture of target protein and nuclear. The immunofluorescence staining was imaged by fluorescence microscopy (Zeiss LSM 800 laser, ×200 magnification), and the mean value of fluorescence intensity was used to perform calculation (*n* = 6). (**B**) The protein expression of EGF, VEGF, MMP-9 and ERα in wound tissue of MLKL^+/+^ and MLKL^−/−^ mice was evaluated by western blot (*n* = 4–6). GAPDH was used as a loading control. Grayscale values were measured using ImageJ software. Littermate control mice were on a C57BL/6J genetic background were utilized in the experiments. Results were expressed as the mean ± SD of multiple independent experiments and analyzed by student t test. *P < 0.05, **P < 0.01, ***P < 0.001. *a. u.* arbitrary unit. Scale label = 20 μm.

The expression levels of growth factors, including epidermal growth factor (EGF), VEGF, estrogen receptor α (ERα), and matrix metalloprotein-9 (MMP-9) in the wound tissue of both MLKL^+/+^ and MLKL^−/−^ mice were measured using immunofluorescence staining and Western blot analysis. The results revealed that the expression levels of EGF, VEGF, ERα, and MMP-9 in the MLKL^−/−^ wound area were lower compared with that in MLKL^+/+^ mice on day 5, 7 and 10 (Fig. 4). These findings suggest that MLKL is involved in regulating the synthesis of these growth factors at skin wound sites.

### MLKL in M1/M2 macrophages regulates the activity of myofibroblasts

**Fig. 5:**
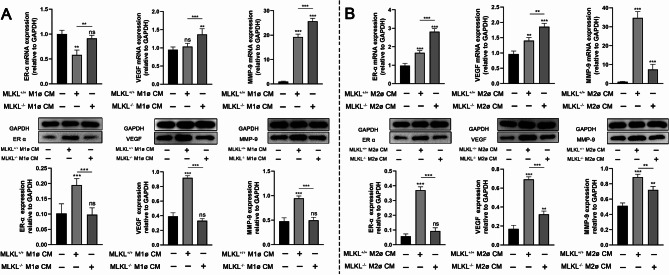
MLKL in macrophages affects the activity of myofibroblasts. (**A**) The mRNA and protein expression of VEGF, MMP-9 and ERα in MLKL^+/+^ myofibroblasts treated with MLKL^+/+^ M1ø CM, or MLKL^−/−^M1ø CM. (**B**) The expression of VEGF, MMP-9 and ERα mRNA and protein in MLKL^+/+^ myofibroblasts treated with MLKL^+/+^ M2ø CM, or MLKL^−/−^M2ø CM. GAPDH was used as a loading control. Grayscale values were measured using ImageJ software. Littermate control mice were on a C57BL/6J genetic background were utilized in the experiments. Results were expressed as the mean ± SD and were analyzed by one-way ANOVA followed by Tukey’s multiple comparisons test (*n* = 4–6). **P < 0.01, ***P < 0.001. *ns* not significant.

To explore the roles of MLKL in M1 macrophages in regulating the activity of myofibroblasts, MLKL^+/+^ and MLKL^−/−^ M1ø CM were co-cultured with MLKL^+/+^ myofibroblasts separately. We found that the inducible effect of MLKL^+/+^ M1ø CM on ERα, VEGF, and MMP-9 protein expression in myofibroblasts was inhibited when treatment was replaced with MLKL^−/−^M1ø CM (Fig. 5A). Similarly, to explore the roles of MLKL in M2 macrophages in regulating the activity of myofibroblasts, MLKL^+/+^ and MLKL^−/−^ M2ø CM were co-cultured with MLKL^+/+^ myofibroblasts separately. We found that the inducible effect of MLKL^+/+^ M2ø CM on ERα, VEGF, and MMP-9 protein expression in myofibroblasts was also inhibited when treatment was replaced with MLKL^−/−^ M2ø CM (Fig. 5B). The mRNA expression is not always consistent with protein expression of target, while these results suggested that MLKL in macrophages regulates the growth factors expression in myofibroblasts.

### MLKL in fibroblasts regulate the activity of M1/M2 macrophages

**Fig. 6:**
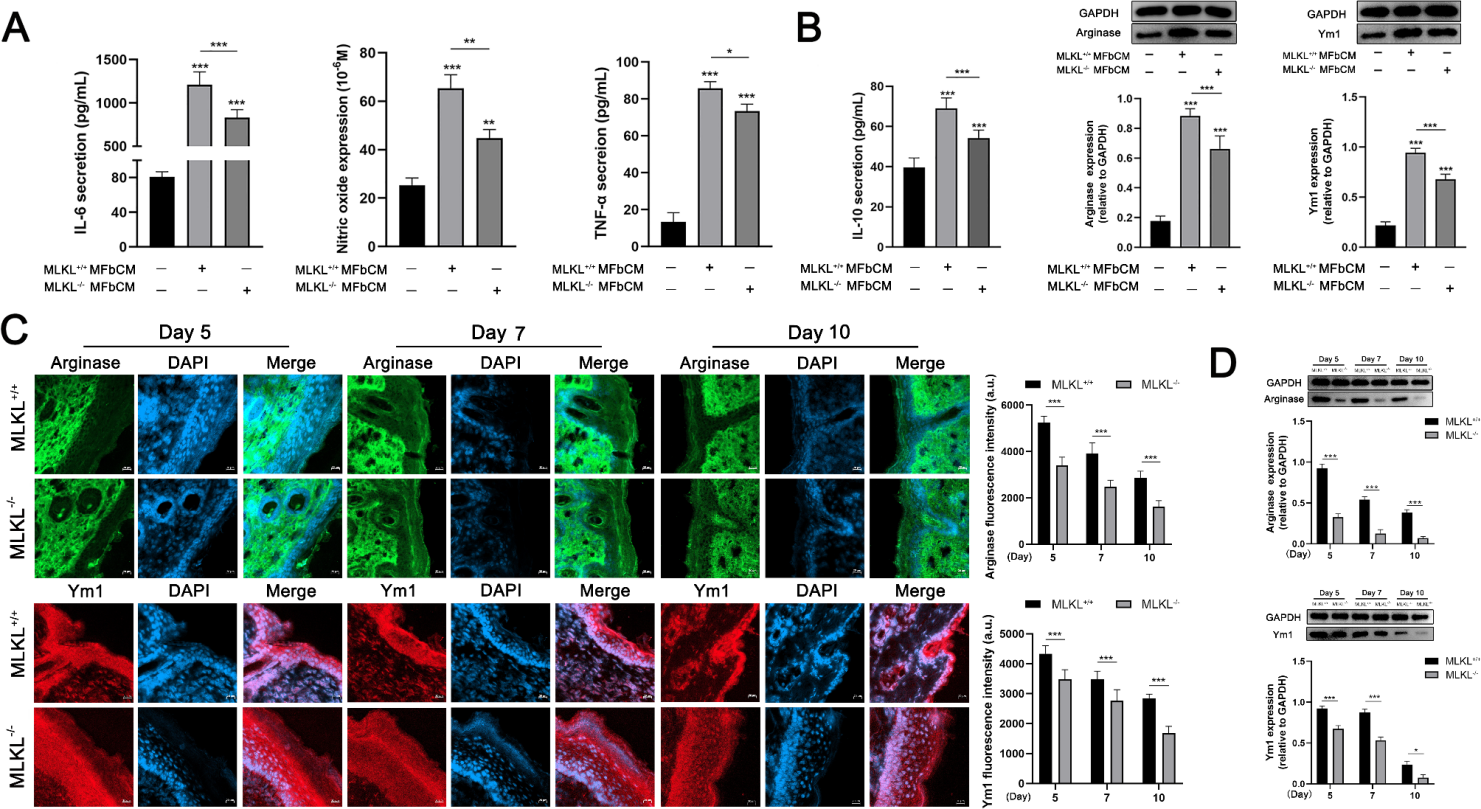
MLKL in myofibroblasts affects the activity of macrophages. (**A**) The secretion of IL-6, NO and TNF-α in MLKL^+/+^ M1ø treated with MLKL^+/+^ MFbCM, or MLKL^−/−^ MFbCM. (**B**) The secretion of IL-10, arginase and Ym1 in MLKL^+/+^ M2ø treated with MLKL^+/+^ MFbCM, or MLKL^−/−^ MFbCM. (**C**)The skin wound tissues of MLKL^+/+^ and MLKL^−/−^ mice were subjected to immunofluorescence staining for arginase (red), Ym1 (green) and nuclear (DAPI, blue) on the 5, 7 and 10th days after wound injury. Merge represents the composite picture of target protein and nuclear. The immunofluorescence staining was imaged by fluorescence microscopy (Zeiss LSM 800 laser, ×200 magnification), and the mean value of fluorescence intensity was used to perform calculation (a. u., arbitrary unit). (**D**) The protein expression of arginase and Ym1 in wound tissues of MLKL^+/+^ and MLKL^−/−^ mice were determined by western blot. GAPDH was used as a loading control. Grayscale values were measured using ImageJ software. Littermate control mice were on a C57BL/6J genetic background were utilized in the experiments. Results were expressed as the mean ± SD of multiple independent experiments and analyzed by Student t test or one-way ANOVA followed by Tukey’s multiple comparisons test (*n* = 4–6). *P < 0.05, **P < 0.01, ***P < 0.001. Scale label = 20 μm.

We individually co-cultured M1 and M2 macrophages in MLKL^+/+^ fibroblast-conditioned medium (MFbCM). The results indicated that MLKL^+/+^ MFbCM induced the synthesis of IL-6, nitric oxide (NO), and TNF-α in M1 macrophages, whereas this inductive effect was inhibited when MLKL^−/−^ MFbCM was used (Fig. 6A). We observed lower expression of arginase and Ym1 at the MLKL^−/−^ skin wound site than at the MLKL^+/+^ wound site (Fig. 6C, D). Additionally, the results showed that MLKL^+/+^ MFbCM induced the synthesis of IL-10, arginase, and Ym1 in M2 macrophages; however, this inductive effect was inhibited when MLKL^−/−^ MFbCM was used (Fig. 6B).

### PGE_2_ is one of the mediators in the cross-talk between macrophages and myofibroblasts

**Fig. 7:**
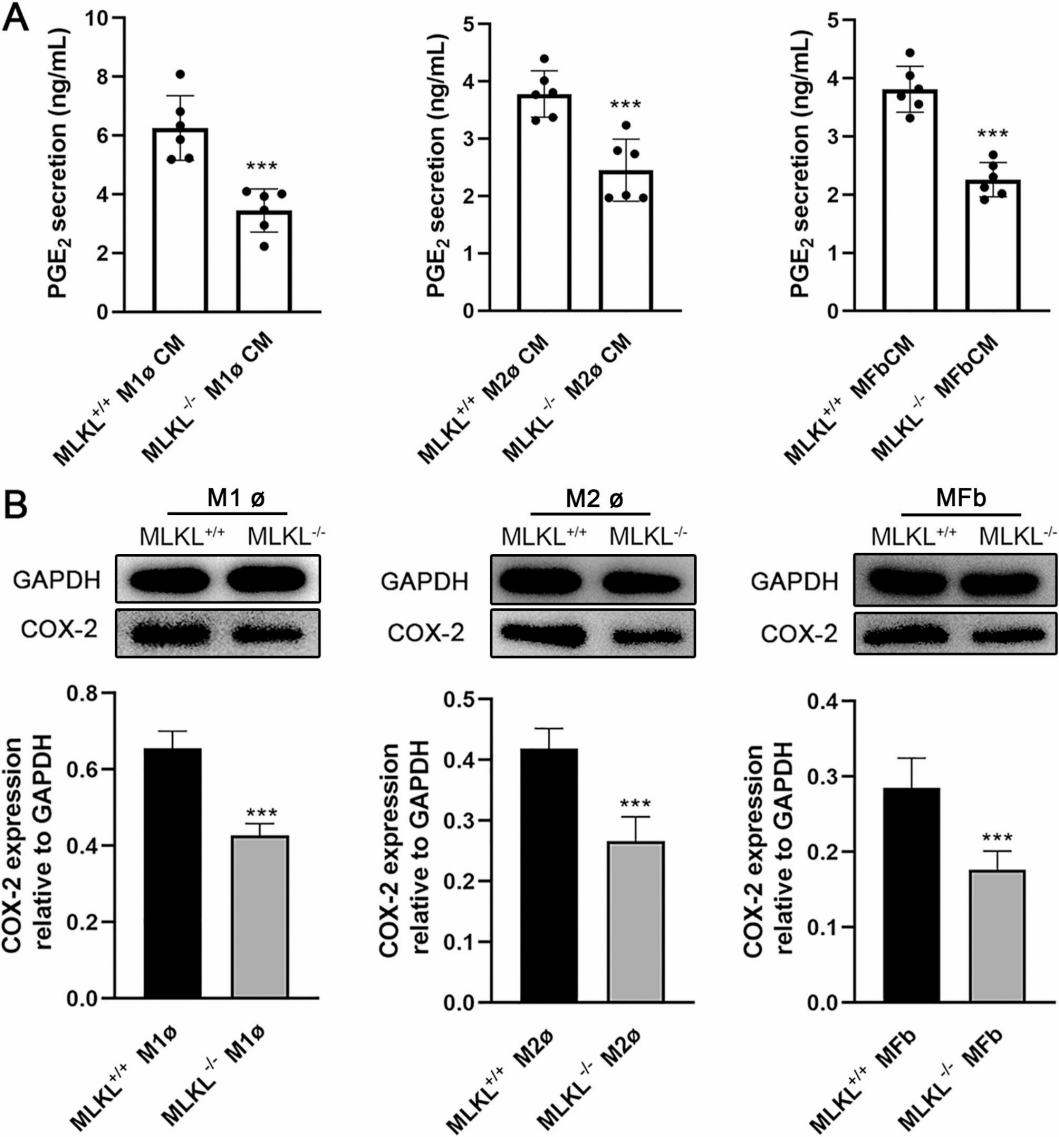
MLKL affects PGE_2_ synthesis in macrophages and myofibroblasts. (**A**) PGE_2_ levels in M1/M2 macrophages- and myofibroblasts-conditioned medium of MLKL^+/+^ and MLKL^−/−^ mice. (**B**) COX-2 protein levels in M1/M2 macrophages and myofibroblasts of MLKL^+/+^ and MLKL^−/−^ mice. GAPDH was used as a loading control. Grayscale values were measured using ImageJ software. Littermate control mice were on a C57BL/6J genetic background were utilized in the experiments. Results were expressed as the mean ± SD and were analyzed by Student t test (*n* = 6). ***P < 0.001.

**Fig. 8:**
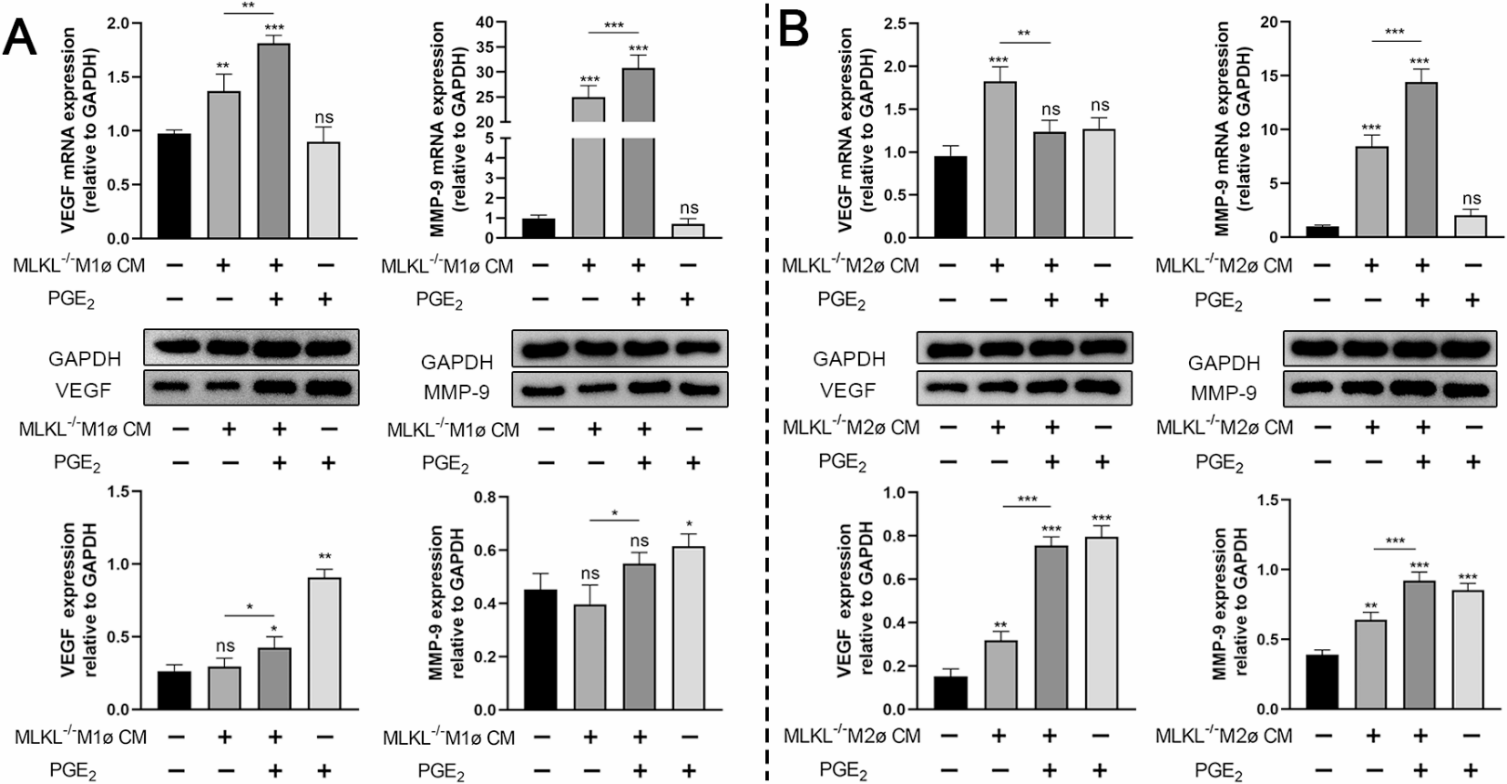
MLKL in macrophages affects myofibroblasts activities through PGE_2_. (**A**) The mRNA and protein expression of VEGF, MMP-9 in MLKL^+/+^ myofibroblasts treated with MLKL^−/−^ M1ø CM or with PGE_2_ supplement. (**B**) The mRNA and protein expression of VEGF, MMP-9 in MLKL^+/+^ myofibroblasts treated with MLKL^−/−^ M2ø CM or with PGE_2_ supplement. GAPDH was used as a loading control. Grayscale values were measured using ImageJ software. Littermate control mice were on a C57BL/6J genetic background were utilized in the experiments. Results were expressed as the mean ± SD and were analyzed by one-way ANOVA followed by Tukey’s multiple comparisons test (*n* = 4–6). *P < 0.05, **P < 0.01, ***P < 0.001. *ns* not significant.

**Fig. 9:**
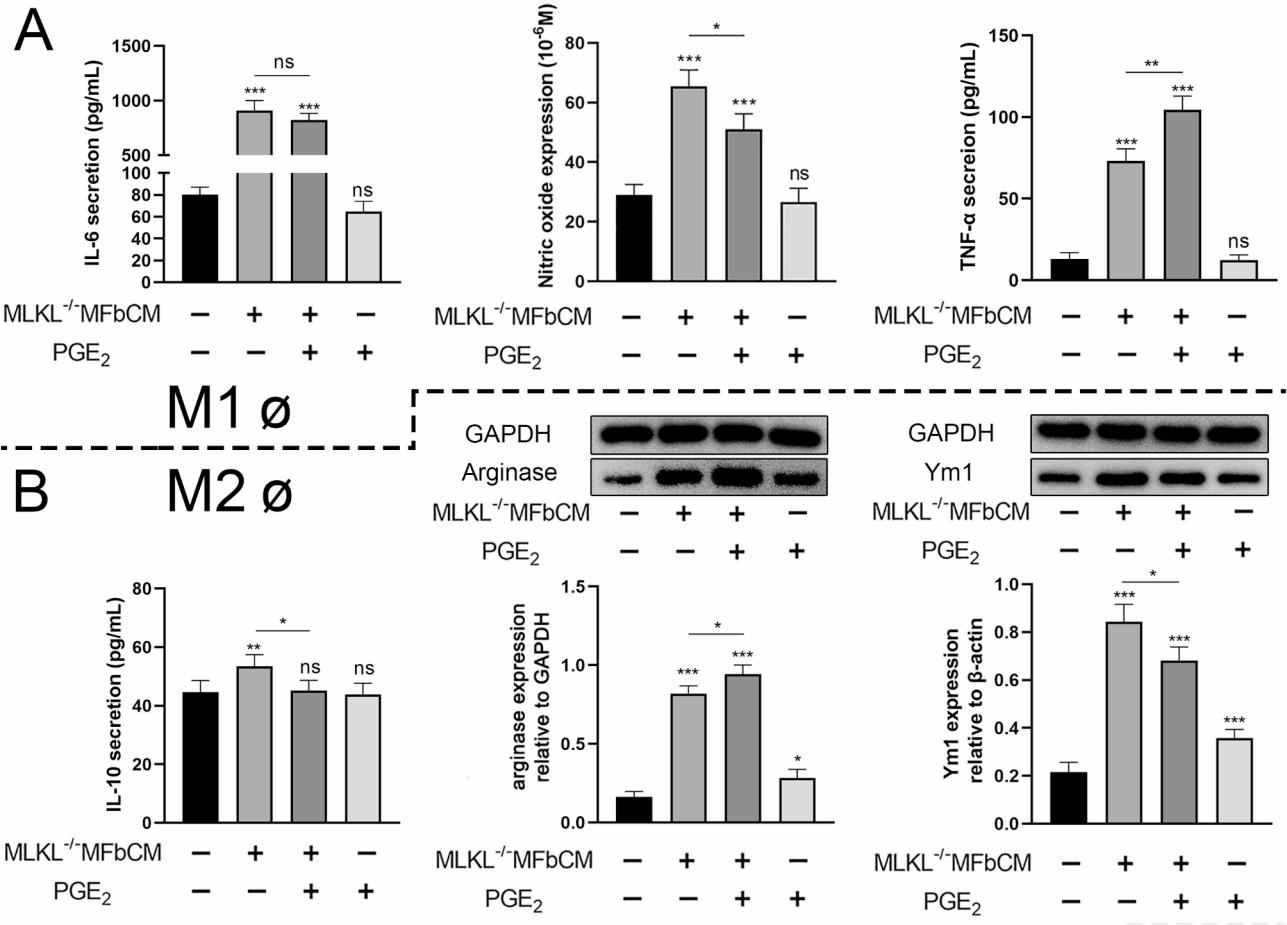
MLKL in myofibroblasts affects macrophages activities through PGE_2_. (**A**) The production of IL-6, NO and TNF-α in MLKL^+/+^ M1 macrophages after MLKL^−/−^ MFbCM treatment or MLKL^−/−^ MFbCM plus PGE_2_ treatment. (**B**) The production of IL-10, arginase and Ym1 in MLKL^+/+^ M2ø after MLKL^−/−^ MFbCM treatment or MLKL^−/−^ MFbCM plus PGE_2_ treatment. GAPDH was used as a loading control. Grayscale values were measured using ImageJ software. Littermate control mice were on a C57BL/6J genetic background were utilized in the experiments. Results were expressed as the mean ± SD and were analyzed by one-way ANOVA followed by Tukey’s multiple comparisons test (*n* = 4–6). *P < 0.05, **P < 0.01, ***P < 0.001. *ns* not significant.

The above-mentioned research indicates that MLKL plays a role in the interaction between macrophages and myofibroblasts. We observed that the expression of cyclooxygenase-2 (COX-2) was lower in MLKL^−/−^ M1/M2 macrophages and myofibroblasts compared to MLKL^+/+^ cells (Fig. 7B). Additionally, the secretion of PGE_2_ was reduced in MLKL^−/−^ M1ø/M2ø CM and MFbCM compared to MLKL^+/+^ CMs (Fig. 7A), suggesting that PGE_2_ might act as an intermediary in the interactions between macrophages and myofibroblasts. To explore this further, we supplemented MLKL^−/−^ MFbCM or M1ø / M2ø CM with exogenous PGE_2_ to match the amount lost in MLKL^−/−^ cells. The results demonstrated that MLKL^−/−^ M1ø CM or M2ø CM supplemented with PGE_2_ enhanced the protein expression of VEGF and MMP-9 in myofibroblasts (Fig. 8A, B). However, when MLKL^−/−^ MFbCM was supplemented with PGE_2_, the effects differed: IL-6 synthesis remained unaffected, NO secretion was further inhibited, and TNF-α was enhanced in M1 macrophages (Fig. 9A). Additionally, MLKL^−/−^ MFbCM supplemented with PGE_2_ decreased IL-10 and Ym1 expression, while enhancing arginase expression in M2 macrophages (Fig. 9B).

## Discussion

This study investigated the role of MLKL in skin wound healing and its interaction with macrophages and fibroblasts. Using MLKL^−/−^ and MLKL^+/+^ mice, research has demonstrated that MLKL deficiency delays wound healing, disrupts inflammatory responses, apoptosis, and impairs tissue repair-associated factors in wound site. Furthermore, this study suggests that MLKL contributes to the bidirectional crosstalk between macrophages and fibroblasts, with PGE_2_ identified as a key mediator. These findings establish the pivotal role of MLKL in orchestrating cellular interactions that are critical for tissue regeneration and highlight its potential as a therapeutic target for improving wound healing outcomes.

The initial damage stimulus induces a conformational change in the cells at the wound site, leading to plasma membrane translocation and lethal permeation of the lipid bilayer. This process results in the release of cellular contents and subsequent activation of an inflammatory response^26^. Pro-inflammatory chemokines and cytokines released by activated macrophages, primarily M1 macrophages, initiate the recruitment and activation of additional immune cells, including neutrophils and bone marrow-derived monocytes, to the site of injury. These immune cells further amplify the inflammatory response to eliminate damaged signals and necrotic cells^16^. RIPK3, a regulator of necroptosis, deficiency significantly delayed wound closure and impaired wound healing, as evidenced by delayed re-epithelialization, angiogenesis, granulation tissue formation, and collagen deposition^5,27^. MLKL, a functional substrate of RIPK3, acts as an adaptor protein for necrosis signal transduction^28^. Both RIPK3 and MLKL overexpression was observed at the skin wound site in MLKL^+/+^ mice (Fig. 1). Additionally, we noticed that three other targets (Sycp2, HK3, and TNF-α) are overexpressed at the wound site throughout the healing process. Sycp2 is a synaptonemal complex protein that is associated with meiosis^29^. HK3 is essential for initiating glycolysis^30^. Both Sycp2 and HK3 may be involved in cellular differentiation related to inflammatory and immune responses^30^. TNF-α is crucial in both apoptosis and necroptosis, and its interaction with MLKL in necroptosis is well-documented^31^. This helps explain the co-overexpression of TNF-α and MLKL observed at wound sites in MLKL^+/+^ mice. In MLKL^−/−^mice, delayed skin wound closure and impaired morphological characteristics throughout the healing process are observed (Fig. 2). The inflammation in wound site is reduced by MLKL deficiency evidenced by the lower concentration of inflammatory cytokines, including PGE_2_, TNF-α, and IL-6 in the wound tissue of MLKL^−/−^ mice compared with that in MLKL^+/+^ mice three days post-injury. Inflammation plays a critical role in the early stages of wound healing and involves both its initiation and resolution phases. Apoptosis is essential for resolving inflammation, particularly through the clearance of neutrophils and other immune cells at the wound site^32^. This process helps transition from the inflammatory to the reparative phase of wound healing^33^. Our findings showed that MLKL deficiency not only reduced inflammation (evidenced by reduced TNF-α, IL-6 and PGE_2_ levels) but also suppressed apoptosis (evidenced by reduced Caspase-3 and P53 and increased Bcl-2) at the wound site. This observation suggests that the reduced inflammation in MLKL-deficient mice is likely due to diminished secretion or release of inflammatory cytokines (potentially related to necroptosis), rather than enhanced apoptosis clearing inflammatory cytokines or DAMPs.

Serum levels of TNF-α and IL-6 were also reduced in MLKL^−/−^ mice, while PGE_2_ levels were elevated in their serum after injury (Fig. 3 and Fig. S1). Local vs. systemic PGE_2_ regulation might help to explain this different secretion pattern in serum and wound site. MLKL plays a critical role in mediating localized inflammatory responses. In its absence, the inflammatory activity at the wound site is likely reduced, leading to subsequent PGE_2_ synthesis locally^34^. Conversely, systemic compensatory mechanisms might lead to elevated PGE_2_ production in other tissues resulting in increased serum PGE_2_ levels^35,36^. Or MLKL deficiency may disrupt the necroptotic process, leading to reduced recruitment of immune cells (contributor to PGE_2_ product) to the wound site^37^, reducing PGE_2_ synthesis locally, while systemic immune activation persists or is enhanced, contributing to elevated serum PGE_2_^34^. Other possibility, such as metabolic difference between MLKL^−/−^ and wild-type mice in PGE_2_ clearance leading accumulation in the serum^38,39^, cannot be excluded well.

As wound healing progresses from the pro-inflammatory to the pro-healing phase, it transitions to the remodeling stage. This stage is marked by a shift in the macrophage phenotype from pro-inflammatory (M1) to anti-inflammatory (M2)^40^. Whether M2 macrophages at injury sites arise from blood monocytes or result from the phenotypic conversion of M1 macrophages remains debatable^11^. These anti-inflammatory macrophages are crucial for the resolution of inflammation and the progression of remodeling. They contribute to the healing process through their regenerative properties by secreting angiogenic and growth factors, cytokines, and chemokines, such as MMPs, VEGF, IL-8, TGF-β, IL-10, and arginase etc., which are essential for recruiting and activating other cells^40,41^. Macrophage during the regenerative phase can directly or indirectly affect wound revascularization, matrix production, and re-epithelialization^42^. Our study revealed that MLKL overexpression was sustained in the wound area during the mid- and late-stages of healing, indicating that MLKL signaling may be important not only in the early inflammatory phase, but also in the later stages of wound repair. We found that MLKL deficiency impairs the expression of growth-related factors including EGF, VEGF, MMP-9, and ERα, suggesting that MLKL plays a role in regulating growth factor expression at the wound site (Fig. 4). The delayed wound regeneration observed in MLKL^−/−^ mice, as evidenced by H&E staining, is consistent with these findings. Specifically, collagen fibers and mature granulation tissue were significantly more abundant in the wounds of MLKL^+/+^ mice than in those of MLKL^−/−^ mice at later stages of healing.

Miscommunication between macrophages and fibroblasts is a critical factor that shifts the balance from physiological repair to pathological fibrosis^15^. Macrophage depletion during wound healing leads to a reduction in fibroblast numbers and that macrophages play a role in scavenging TGF-β, a key regulator of fibroblast/myofibroblast activation^16^. Supernatants from MLKL^+/+^ M1 macrophages show enhanced expression of VEGF and MMP-9 in fibroblasts. This effect was partially inhibited when supernatants from MLKL^−/−^ M1 macrophages were used, indicating that MLKL in macrophages regulates myofibroblast activity. Additionally, PGE_2_ synthesis is increased at wound site according to our and previous study^43^. As stated in introduction, we hypothesized that PGE_2_ is a potential mediator in the cross-talk between M1 macrophages and myofibroblasts. It was found that COX-2 (the upstream enzyme of PGE_2_ synthesis) expression and PGE_2_ levels in MLKL^−/−^ M1 macrophages were lower than that in MLKL^+/+^ cells (Fig. 7). To investigate this further, we replenished PGE_2_ in a co-culture system of MLKL^−/−^ M1 CM and myofibroblasts. This restoration of PGE_2_ levels led to a recovery in the expression patterns of VEGF and MMP-9 in myofibroblasts (Fig. 8A). Conversely, MLKL in myofibroblasts was found to regulate the synthesis of IL-6, NO, and TNF-α in M1 macrophages through PGE_2_ (Fig. 9A). Totable, NO and TNF-α secretion pattern was different after PGE_2_ supplement, suggesting that the roles of PGE_2_ is complex in regulating inflammatory mediator synthesis in interaction between macrophages and fibroblasts. This can be explained by the PGE_2_ has both anti- and pro-inflammatory effects dependent on the timing of its production and concentration^44–47^.These findings suggest that PGE_2_ may act as an intermediary in MLKL-mediated interactions between M1 macrophages and myofibroblasts. Zeinab et al. found that PGE_2_ secretion levels in MLKL^−/−^ macrophages was lower than that in wild-type after stimulation^48^. Regarding the reduced PGE_2_ concentration in the supernatants from MLKL^−/−^ macrophages (both M1 and M2) and myofibroblasts in this study. MLKL deficiency might directly reduce PGE_2_ synthesis in these cells, or it might delay membranolysis, trapping PGE_2_ within the cells. Our data, particularly the reduced COX-2 expression in MLKL^−/−^ cells, support that MLKL deficiency might directly reduce PGE_2_ synthesis in these cells. Taken together, MLKL may contributes to inflammatory microenvironment at the wound site by modulating the interaction between M1 macrophages and fibroblasts.

M2 macrophages produce anti-inflammatory cytokines and growth factors that mitigate inflammation, activate fibroblasts, and promote angiogenesis and wound contraction^16^. Ablation of macrophages reduces myofibroblast numbers in the wound bed, impairs myofibroblast function, and hinders wound healing^13,14^. Prolonged pathological stimulation, leading to an unresolved crosstalk between M2 macrophages and fibroblasts, often results in pathological fibrosis or chronic wounds in several tissues^49^. Thus, understanding the interactions between M2 macrophages and fibroblasts is essential. We observed that the expression of growth factors, EGF, VEGF, and MMP-9, significantly increased at the late stage of wound healing; however, their levels were lower in MLKL^−/−^ wound sites compared with those in MLKL^+/+^ sites. MLKL in M2 macrophages has been shown to regulate VEGF and MMP-9 expression in myofibroblasts, potentially explaining the reduced growth factor expression observed at the wound site in MLKL-deficient conditions. Additionally, MLKL in fibroblasts regulated the expression of arginase, Ym1, and IL-10 in M2 macrophages, indicating that MLKL in fibroblasts may influence M2 macrophage activity (Fig. 6). Ym1 and arginase, which are predominantly expressed in macrophages at the sites of injury, play crucial roles in wound healing and resolution^42^. Arginase contributes to wound healing through the production of L-ornithine; its absence impairs cutaneous wound healing, while its overproduction contributes to fibrosis^50^. IL-10 promotes the differentiation of macrophages into pro-regenerative phenotypes^51^. PGE_2_ released from myofibroblasts, along with its receptor agonist, enhances arginase activity in M2 macrophages, further supporting the role of PGE_2_ in regulating M2 macrophage activity^20^. We also found reduced COX-2 and PGE_2_ synthesis in MLKL^−/−^ M2 macrophages. Similar to its role in the interaction between M1 macrophages and myofibroblasts, PGE_2_ is also involved in the interaction between M2 macrophages and myofibroblasts (Figs. 8B and 9B). These findings highlight the importance of MLKL and PGE_2_ in mediating complex interactions between macrophages and fibroblasts during wound healing.

While the mouse model is widely adopted due to its convenience and the availability of genetic tools, we acknowledge its significant differences compared to human skin. For instance, mouse skin is thinner and primarily heals through contraction, whereas human skin relies more on granulation tissue formation and re-epithelialization. We would like to emphasize that while our study reveals the role of MLKL in wound healing and macrophage-fibroblast crosstalk, extrapolating these findings to human wound healing should be approached with caution and supported by validation in alternative models, including porcine or humanized skin models. Furthermore, how MLKL modulates the inflammatory response at the wound site remains unaddressed. We proposed that, in MLKL-deficient mice, impaired necroptosis could lead to decreased DAMP release, thereby attenuating the inflammatory response. There is another possibility cannot be excluded, in which active MLKL directly regulate immune cells recruitment and intrinsic inflammatory mediator synthesis. Such as, MLKL has been shown to trigger the NLRP3 inflammasome in a cell-intrinsic manner, leading to the maturation and release of pro-inflammatory cytokines like IL-1β^52^. In the absence of MLKL, this pathway may be less active, contributing to reduced inflammation at the wound site. We recognize that these proposed mechanisms are speculative and warrant further investigation. Future studies should aim to elucidate the precise pathways through which MLKL influences inflammatory mediator release in wound site. Understanding these mechanisms could provide deeper insights into the role of MLKL in inflammation and tissue regeneration during skin wound healing. In addition, incorporating transepidermal water loss (TEWL) measurements in future experiments would provide a more objective evaluation of wound healing progress.

In conclusion, this study highlights the pivotal role of MLKL in skin wound healing process, demonstrating its involvement in both inflammatory responses and bidirectional interaction between macrophages and fibroblasts. Using a murine skin excision model, we observed delayed wound closure and impaired tissue repair in MLKL-deficient mice, with a reduction in key inflammatory mediators, growth factors, and structural tissue components. Furthermore, we identified PGE_2_ as a crucial intermediary in MLKL-mediated crosstalk between macrophages and fibroblasts, underscoring its importance in tissue repair dynamics.

## Material & methods

### Animals

The experiments were performed under the guidance of the Regulations for the Administration of Affairs Concerning Experimental Animals in China (2017). The Animal Welfare and Research Ethics Committee of the university approved the experimental design (Approval ID: NND2022021). The MLKL^+/+^ mice were provided by the Model Animal Research Center of Nanjing University (Nanjing, China). MLKL^−/−^ mice on a C57BL/6J background were obtained from Xiamen University, and their knockout status was further confirmed by assessing MLKL expression. Both male and female mice were used for the experiments. Littermate control mice were on a C57BL/6J genetic background were utilized in all experiments. The mice were housed in a clean environment (12 h day/night cycles, 22–24℃ and 50% humidity) and supplied with unlimited food and water. Eight-week-old mice (25–30 g) were used in all the experiments. This study was conducted in accordance with the ARRIVE guidelines (https://arriveguidelines.org).

### Experimental design

In in vivo study, transcriptome sequencing, and analysis of wound sites from MLKL^+/+^ mice were conducted to identify potential genes involved in skin wound healing. To investigate the roles of MLKL in this process, we created skin wounds in both MLKL^+/+^ and MLKL^−/−^ mice. The wound healing ratio, morphological characteristics, expression of tissue repair-related factors, inflammatory mediators, and apoptosis-related factors at the wound site were monitored throughout the healing process using H&E and immunofluorescence staining.

In in vitro study, M1/M2 macrophages and myofibroblasts were used to investigate the role of MLKL in interactions between these cell types. To explore the roles of MLKL in M1/M2 macrophages in regulating myofibroblast activity, We co-cultured MLKL^+/+^ and MLKL^−/−^ M1ø CM or M2ø CM with MLKL^+/+^ myofibroblasts as previously described^20^. The expression of ERα, VEGF, and MMP-9 in myofibroblasts was assessed. To investigate the roles of MLKL in myofibroblasts on M1/M2 macrophages, we co-cultured MLKL^+/+^ and MLKL^−/−^ MFbCM with MLKL^+/+^ M1/M2 macrophages separately. The expression of IL-6, NO, and TNF-α in M1 macrophages was measured, as well as IL-10, arginase, and Ym1 in M2 macrophages. Finally, the role of PGE_2_ in the MLKL-mediated interactions between macrophages and myofibroblasts were investigated. An abstract of the experimental design is attached (Supplementary Material, Fig. S2).

### Cutaneous wound model

A circular excisional wound was directly created on the dorsal skin of MLKL^+/+^ and MLKL^−/−^ mice. Mice were anesthetized by isoflurane inhalation. The dorsum was shaved and sterilized with 75% alcohol. A 6-mm diameter skin biopsy punch was used to create an excision wound extending to the fascia. Wound closure progression was measured daily until day 14. On days 1, 3, 5, 7, 10, 12, and 14 after wound injury, the mice were sacrificed under anesthesia, and skin samples were collected from the entire wound site (including the scab and epithelial margins). The skin samples were fixed and stored in 10% formalin for histological analysis, frozen in liquid nitrogen, and stored at -80 °C for molecular experiments. In this study, littermate mice were used as experimental subjects to minimize variability due to genetic and environmental differences. All mice used in the experiments were 8 weeks old, weighing between 25 and 30 g, ensuring consistency in age and growth stage. To prevent disturbance of the wounds, all mice were housed individually in ventilated cages after injury.

### Transcriptome analysis

Total mRNA was extracted using the TRIzol reagent (Invitrogen, CA, USA). Libraries were constructed using the VAHTS Universal V6 RNA-seq Library Prep Kit. Transcriptome sequencing and analysis were performed by OE Biotech Co., Ltd. (Shanghai, China). Differentially expressed genes (DEGs) were identified using DESeq2 and screened under the criteria of log2 |fold change| > 1.5 and *P* < 0.05. The identified DEGs were subjected to Gene Ontology (GO) and Kyoto Encyclopedia of Genes and Genomes (KEGG) pathway enrichment analyses using OECloud tools (https://cloud.oebiotech.com/task/). Significant enrichment was defined as a *P* < 0.05 and the top 30 enrichment results were presented.

### Wound healing rate determination

The wound healing rates at 1, 3, 5, 7, 10, 12, and 14 d were calculated by comparing the wound surface area to the original wound area. The percentage of wound closure was determined using the formula: (Area Day 1 - Area Day X)/Area Day 1 × 100%. Digital images of wound surfaces were captured using a SONY Alpha 6400 camera. The wound area was quantified using the ImageJ software (National Institutes of Health, MD, USA).

### Hematoxylin and eosin (H&E) staining

The skin tissue samples fixed in 10% formalin were cut into 2-µm thick sections. The sections were dehydrated using a graded series of ethanol (100, 75, 50, and 25%), embedded in paraffin, and stained with H&E. Images of the stained sections were acquired using an Axio Scan.Z1 slide scanner (Zeiss, Oberkochen, Germany).

### Immunofluorescence staining

The excised dorsal tissue samples from MLKL^+/+^ and MLKL^−/−^ mice were frozen in liquid nitrogen and stored at -80 °C. Subsequently, the tissues were thawed and embedded in Tissue-Tek OCT compound (Sakura Finetek, CA, USA), and 6-µm thick cryosections were prepared using a freezing microtome. The sections were fixed in cold acetone for 10 min, washed with cold endotoxin-free phosphate-buffered saline (PBS) containing 0.25% Tween-20, and blocked for 1 h with 3% bovine serum albumin. Primary antibodies were then added and the sections were incubated in the dark for 14 h at 4 °C. After incubation, the slides were washed three times in PBS with 0.25% Tween-20 and incubated with fluorescently labeled secondary antibodies for 1 h at room temperature (24–26 ℃). DAPI (4’,6-diamidino-2-phenylindole) was used for nuclear counterstaining. We randomly selected three visual fields per section around the wound from each group (*n* = 3–5 mice/group) and confocal microscopy (LSM 800, Zeiss, Oberkochen, Germany) provided the fluorescence intensity value as the relative fluorescence intensity (a.u.). In order to ensure the consistency and comparability of immunofluorescence background, we set the same values of black and white in the software for the fluorescence images presented with the same factors so that the visual data we presented was as reliable and interpretable as possible. The details of the antibodies used are listed in (Table 2).

**Table 2:** Antibodies used in Immunofluorescence staining.

Name	Description	Concentration	Company	Cat no.
p53	Mouse monoclonal	1:2000	Cell signaling technology	2524T
Bcl-2	Mouse monoclonal	1:50	Cell signaling technology	15,071
Caspase-3	Rabbit polyclonal	1:1000	abcam	ab49822
ERα	Mouse monoclonal	1:500	GeneTex	GTX13538
MMP-9	Rabbit polyclonal	20ug/ml	Novus	NBP2-41233
EGF	Rabbit monoclonal	1:500	abcam	ab184266
VEGF	Mouse monoclonal	200ug/ml	Santa	SC-7269
arginase	Mouse monoclonal	1:1000	Santa	SC-47,715
Ym1	Rabbit polyclonal	1:50	Stemcell technologies	60,130
Dnk pAb to Rat IgG	Alexa Fluor^®^ 488	1:1000	abcam	ab150153
Goat pAb to Rb IgG	Alexa Fluor^®^ 647	1:1000	abcam	ab150079

*p53* tumor protein 53, *Bcl-2* B-cell lymphoma 2, *Caspase-3* cysteinyl aspartate specific proteinase 3, *ERα* estrogen receptors α, *MMP-9* matrix metalloproteinase-9, *EGF* epidermal growth factor, *VEGF* vascular endothelial growth factor, *Ym1* chitinase-3-like protein 1, *Dnk* donkey, *pAb* polyclonal antibody, *Rb* rabbit.

### Isolation and culture of bone marrow-derived macrophages (BMDMs)

The BMDMs were isolated from murine femurs and tibias, cultured in RPMI 1640 medium supplemented with 20% fetal bovine serum (FBS; Excell Bio, Shanghai, China), 1.2% glutamine (Thermo Fisher Scientific, Rockford, IL, USA), 2.4% penicillin-streptomycin (Gibco, USA), combined with 20 ng/mL macrophage colony-stimulating factor (M-CSF; PeproTech, NJ, USA) at a density of 5 × 10^6^ cells in a six-well plate. The culture medium was replaced every 24 h for up to 5 days. The BMDMs were treated with 1 µg/mL of lipopolysaccharide (LPS) (PeproTech) for 24 h to differentiate into M1 macrophages. After 24 h stimulation, LPS was removed, the cells were rinsed twice with PBS and cultured in RPMI 1640 medium supplemented with 20% FBS for 24 h. The BMDMs were treated with 20 ng/mL IL-4 in combination with 20 ng/mL IL-13 (PeproTech) for 48 h to induce their differentiation into M2 macrophages. After 48 h stimulation, IL-4 and IL-13 were removed, the cells were rinsed twice with PBS and cultured in RPMI 1640 medium supplemented with 20% FBS for 24 h. M1 and M2 macrophages were identified using flow cytometry (Supplementary Material, Fig. S3). M1ø and M2ø CMs were collected for further experiments. The BMDMs were isolated from 8-week-old littermate mice of both sexes, weighing 25–30 g. This approach ensured consistency in cellular assays and minimized potential variability stemming from differences in genetic background or environmental factors.

### Mice skin myofibroblasts cultivation in vitro

The fibroblasts were isolated from MLKL^+/+^ and MLKL^−/−^ mice. In brief, the ear explants were minced and incubated in 0.25% trypsin-EDTA (Hyclone, UT, USA) for 1 h at 37 °C to remove the epidermis. The explants were placed in tissue culture plates and the migration of fibroblasts from tissue explants to the dishes was observed within 2–5 days. After 2 weeks, tissue explants were removed, and fibroblasts were re-seeded at a density of 1 × 10^5^ cells in a six-well plate and cultured in DMEM/F12 medium supplemented with 10% FBS and 2% penicillin-streptomycin combined with 100 pg/mL TGF-β (PeproTech). The medium was replaced every 24 h until day 5, when the fibroblasts were induced to differentiate into myofibroblasts. The following day, TGF-β was removed, the cells were rinsed twice with PBS and cultured in DMEM/F12 supplemented with 10% FBS for 24 h. The myofibroblasts were identified by α-smooth muscle actin (SMA) expression using an immunofluorescence assay (Supplementary Material, Fig. S4). MFbCM was collected for further experiments. The fibroblasts were isolated from 8-week-old littermate mice of both sexes, weighing 25–30 g. This approach ensured consistency in cellular assays and minimized potential variability stemming from differences in genetic background or environmental factors.

### Western blot (WB) analysis

For total cellular protein extraction, the cells were treated with Mammalian Protein Extraction Reagent (Thermo Fisher Scientific). Protein concentrations were quantified using the BCA assay kit (Thermo Scientific). For WB, 10 µg of total protein per lane were resolved using sodium dodecyl sulfate-polyacrylamide gel electrophoresis (SDS-PAGE) and subsequently transferred to polyvinylidene difluoride membranes. Protein bands were visualized using a Chemiluminescent Substrate (Thermo Fisher Scientific). Grayscale values were quantified using ImageJ (National Institutes of Health). The primary antibodies used are listed in (Table 3).

**Table 3:** Antibodies used in Western blot.

Name	Description	KDa	Concentration	Company	Cat no.
ERα	Mouse monoclonal	71.4 kDa	1:250	GeneTex	GTX13538
MMP-9	Rabbit polyclonal	73 kDa	1 ug/mL	Novus	NBP2-41233
VEGF	Rabbit polyclonal	42 kDa	1 ug/mL	Novus	NB100-2381
arginase	Mouse monoclonal	35 kDa	1:200	Santa	SC-47,715
Ym1	Rabbit polyclonal	45 kDa	1:1000	Stemcell technologies	60,130
GAPDH	Rabbit polyclonal	37 kDa	1:5000	Affinity	AF7021
COX-2	Rabbit monoclonal	70 kDa	1:1000	Cell signaling technology	D5H5
MLKL	Rabbit polyclonal	54 kDa	1:1000	Affinity biosciences	DF7412
Caspase-3	Rabbit polyclonal	17 kDa	1:500	abcam	ab49822
Bcl-2	Mouse monoclonal	26 kDa	1:1000	Cell signaling technology	15,071
p53	Mouse monoclonal	53 kDa	1:1000	Cell signaling technology	2524T
EGF	Rabbit monoclonal	133 kDa	1:1000	abcam	ab184266
Goat Anti-Rabbit IgG	(H + L) HRP	/	1:5000	Affinity biosciences	S0001
Goat Anti-Mouse IgG	(H + L) HRP	/	1:5000	Affinity biosciences	S0002

*ERα* estrogen receptors α, *MMP-9* matrix metalloproteinase-9, *VEGF* vascular endothelial growth factor, *Ym1* chitinase-3-like Protein 1, *COX-2* Cyclooxygenase-2, *MLKL* mixed lineage kinase domain-like protein, *Caspase-3* cysteinyl aspartate specific proteinase 3, *Bcl-2* B-cell lymphoma 2, *p53* tumor protein 53, *EGF* epidermal growth factor.

### Enzyme-linked immunosorbent assay (ELISA) analysis

The harvested wound tissue (0.02–0.04 g) was cut into pieces, homogenized, and lysed with T-PERTM Tissue Protein Extraction Reagent (Thermo Fisher Scientific). Blood was obtained from the eyeballs and centrifuged at 3000 × g for 10 min to extract the serum. The concentrations of IL-6 (Biolegend, CA, USA), TNF-α (Biolegend), and PGE_2_ (Cayman Chemical, MI, USA) were measured in the tissue extracts and serum. The concentration of IL-6, TNF-α, IL-10 (Invitrogen), and NO (Beyotime, Shanghai, China) in the supernatants of cultured macrophages was measured according to the manufacturer’s instructions.

### Real-time RT-PCR analysis

Total mRNA was extracted from the cultured cells using a Total RNA Miniprep Kit (Axygen, California, USA). The extracted RNA was reverse transcribed into cDNA using a cDNA Reverse Transcription Kit (Vazyme, Nanjing, China). Real-time PCR was conducted using an ABI QuantStudio 7 (Thermo Fisher Scientific). The PCR protocol was as follows: initial denaturation for 30 s at 95 °C, followed by 35 cycles of 5 s at 95 °C (denaturation), 34 s at 60 °C (annealing), and 20 s at 72 °C (elongation). GAPDH served as an internal control. Results are presented as 2^−ΔΔCt^ (where ΔΔCt = ΔCt – ΔCt control and ΔCt = Ct target -Ct GAPDH). The primers used for RT-PCR are listed in (Table 4).

**Table 4:** Primers used in this study.

Accession no.	Gene name	Primer sequence
NM_008084.4	GAPDH	Forward:5’ -AGGTCGGTGTGAACGGATTTG-3’ Reverse:3’ -GGGGTCGTTGATGGCAACA-5’’
NM_007956.5	ERα	Forward:5’- TTCTCCCTTTGCTACGTCAC-3’ Reverse:3’- ATCGCTTTGTCAACGACTTC-5’
NM_013599.5	MMP-9	Forward:5’- CGCCTTGGTGTAGCACAACA-3’ Reverse:3’- ACAGGGTTTGCCTTCTCCGTT-5’
NM_009505.4	VEGF	Forward:5’- CTGTAACGATGAAGCCCTGGAG-3’ Reverse:3’- TGGTGAGGTTTGATCCGCAT-5’
NM_029005.3	MLKL	Forward:5’- TATGTCTCCCCTGAGAGACTGAAAA-3’ Reverse:3’- TTCCCAGAGTACAATTCCAAAGCTA-5’

*ERα* estrogen receptors α, *MMP-9* matrix metalloproteinase-9, *VEGF* vascular endothelial growth factor, *MLKL* mixed lineage kinase domain-like protein.

### Data analysis

All data were analyzed using GraphPad Prism 8 (GraphPad Software Inc., USA) and expressed as mean ± standard deviation (SD). Statistical significance was evaluated using one-way analysis of variance (ANOVA) followed by Tukey’s multiple-comparison test or Student t test. *P* < 0.05 were considered statistically significant.

## Electronic supplementary material

Below is the link to the electronic supplementary material.

Supplementary Material 1

## Data Availability

The data for this study are available by contacting the corresponding authors upon reasonable request. The sequencing data of this study are openly available in GenBank of NCBI (Accession No. GSE273056).
