# Supplementary material for: MLKL is involved in the regulation of skin wound healing and interplay between macrophages and myofibroblasts in mice
Source: Sci Rep. 2025 Apr 19;15:13612. doi: 10.1038/s41598-025-97729-2 (PMC12009362; doi:10.1038/s41598-025-97729-2)
Supplement: Supplementary file 1 — Supplementary Material 1 [file 41598_2025_97729_MOESM1_ESM.docx]

**Supplemental Materials**

**
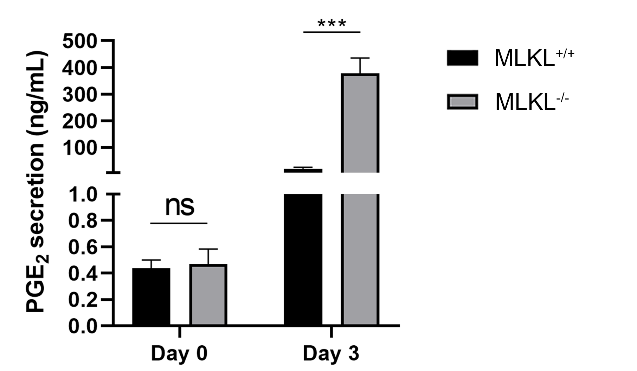
**

**Figure. S1.** The PGE_2_ serum levels

**
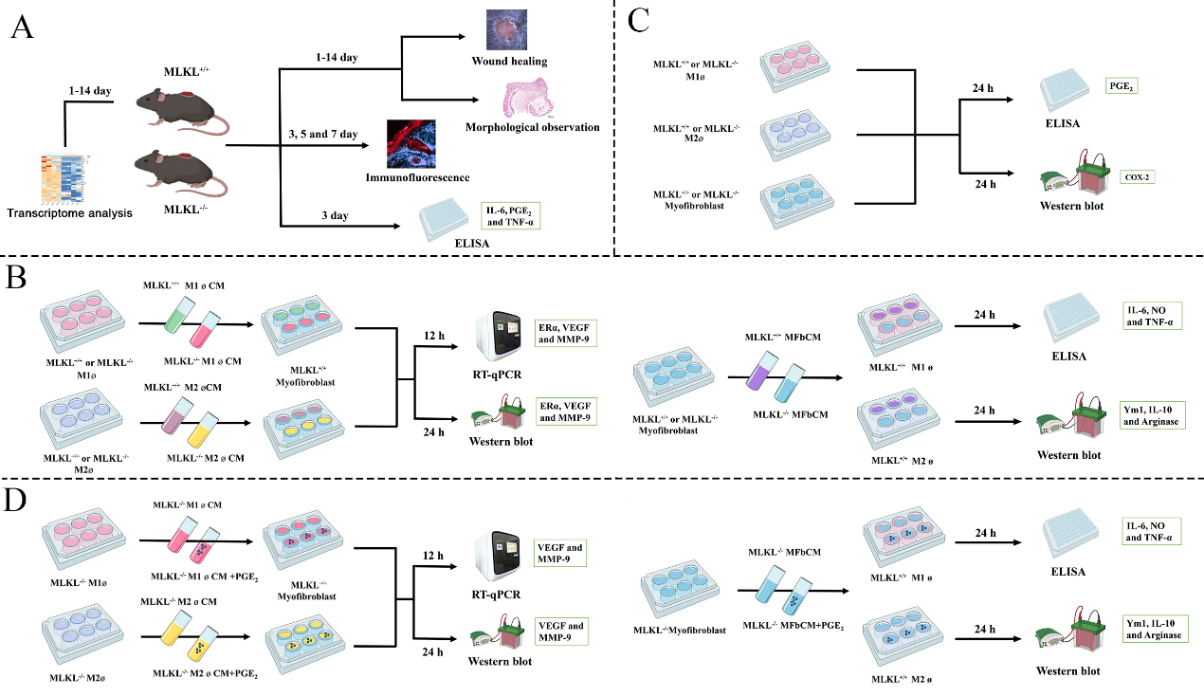
**

**Figure. S2.** Abstract graph of experimental design

**
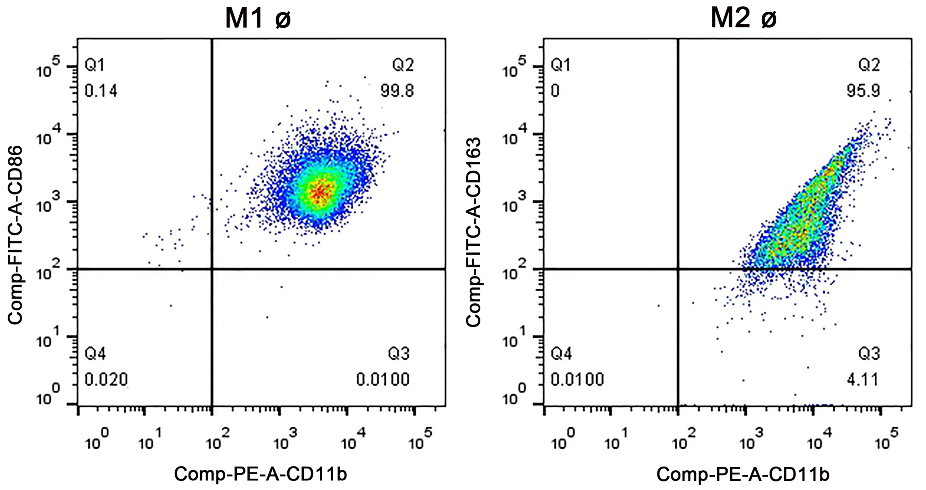
**

**Figure. S3. Identification of cultured M1/M2 macrophages:** M1 macrophages were determined by flow cytometry to be 99.8 % CD86^+^ and CD11b^+^ (M1ø). M2 macrophages were determined by flow cytometry to be 95.9 % CD163^+^ and CD11b^+^ (M2ø).

**
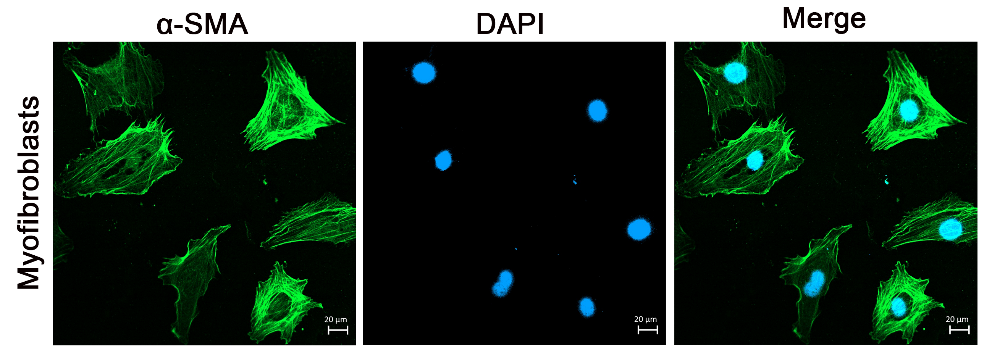
**

**Figure. S4. Identification of cultured myofibroblasts:** Myofibroblast was subjected to immunofluorescence staining for myofibroblast marker α-SMA (green), and nuclear (DAPI, blue). The immunofluorescence staining was imaged by fluorescence microscopy (Zeiss LSM 800 laser, ×400 magnification). Scale label = 20 μm.

**original gels**

All these images exhibited in this presentation were captured using a UVP ChemStudio PLUS system (Analytik Jena, Jena, Germany)

**Full scan of the entire original gel for Figure 1**


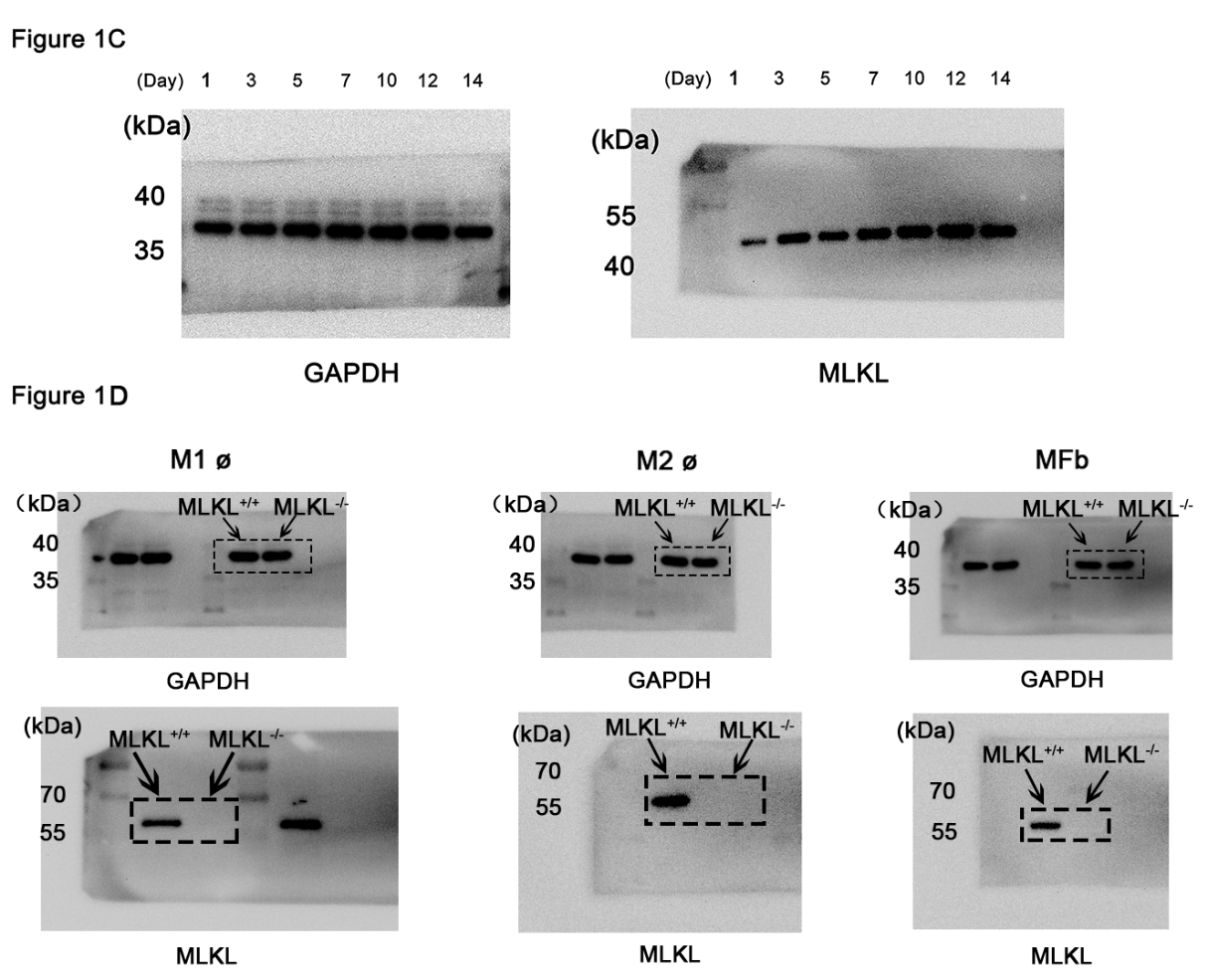


**Full scan of the entire original gel for Figure 3B**





**Full scan of the entire original gel for Figure 4B**





**Full scan of the entire original gel for Figure 5A**





**Full scan of the entire original gel for Figure 5B**

**

**

**Full scan of the entire original gel for Figure 6**

**

**

**Full scan of the entire original gel for Figure 7**





**Full scan of the entire original gel for Figure 8**





**Full scan of the entire original gel for Figure 9B**
